# Interstitial pneumonia disease induced by osimertinib combined with savolitinib targeted therapy in a lung cancer patient: A case report

**DOI:** 10.1097/MD.0000000000036208

**Published:** 2024-01-05

**Authors:** Yikuan Shen, Songgao Lou, Jiansong Zhang

**Affiliations:** a Department of Medicine, Hangzhou Normal University, Hangzhou, Zhejiang, China; b Department of pharmacy, Shengzhou Hospital of Traditional Chinese Medicine, Shaoxing, Zhejiang, China; c Intensive Care Unit, Shengzhou Hospital of Traditional Chinese Medicine, Shaoxing, Zhejiang, China.

**Keywords:** case report, EGFR-TKI, interstitial pneumonia, lung cancer, MET-TKI, targeted therapy

## Abstract

**Rationale:**

MET-TKI is a late-stage treatment for drug-resistant NSCLC that has been marketed in recent years, and interstitial lung disease may be a rare adverse reaction. This case reports the development of interstitial lung disease in a patient with advanced lung cancer who developed during treatment with savolitinib after resistance to osimertinib.

**Patient concerns:**

A 74-year-old female diagnosed with lung adenocarcinoma was referred to our hospital with chest tightness and shortness of breath following treatment with osimertinib plus savolitinib.

**Diagnose:**

Chest CT of the patient shows interstitial changes in both lungs, and drug-related interstitial lung disease is considered in the context of the patient previous condition.

**Interventions:**

The patient is treated with methods such as glucocorticoids, anti-infection, and mechanical ventilation.

**Outcomes:**

At the 1-year follow-up visit, the patient condition of interstitial lung disease was relatively stable. The patient has passed away due to tumor progression.

**Lessons:**

This case reported interstitial lung disease following osimertinib plus savolitinib. This suggests that healthcare providers should be aware of early symptoms of interstitial lung disease during treatment and treat them appropriately to prevent symptoms from worsening.

## 1. Introduction

Lung cancer is a common disease with increasing morbidity and mortality worldwide.^[1]^ According to China national statistics, there are about 631,000 lung cancer deaths every year, and Chinese lung cancer patients have the characteristics of high epidermal growth factor receptor (EGFR) mutations.^[2]^ In patients with advanced NSCLC with EGFR-sensitive mutations, EGFR-TKIs significantly improved objective response rate and progression-free survival compared with conventional chemotherapy.^[3]^ METex14 skipping mutations occur in approximately 3–4% of lung adenocarcinoma patients,^[4]^ and 5%-20% in EGFR-TKIs-resistant patients.^[5]^ Savolitinib, was conditionally approved in China for advanced NSCLC with METex14 jumping mutations,^[6]^ and savolitinib as a late-line treatment for patients with MET14 exon-skipping mutation NSCLC had an objective response rate of 49.2% and progression-free survival of 6 months.^[7]^ Common adverse reactions of MET-TKI are peripheral edema, nausea, elevated alanine transaminase, elevated aspartate transaminase, vomiting, hypoalbuminemia, and decreased appetite; interstitial lung disease is a rare adverse reaction.^[8]^

### 1.1. Consent statement

The patient and the patient next of kin have provided informed consent for publication of this case report and accompanying images.

## 2. Case presentation

**Figure 1.**
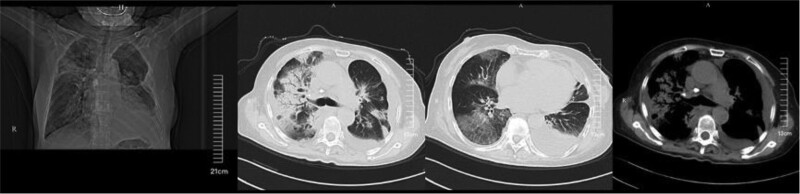
Chest CT before admission. CT = computed tomography.

The patient was a 74-year-old Asian female. In August 2019, the patient was diagnosed with adenocarcinoma of the left lung with bone metastasis in the other hospital, and took EGFR-TKI such as gefitinib and eclitinib. The patient started osimertinib 80 mg/day 1 year ago due to drug resistance. Osimertinib was considered to cause Torsades de pointes 9 months ago, so it was discontinued, but 8 months ago reexamination found lung tumor progression, so osimertinib 80 mg/day was continued under close potassium monitoring and oral potassium supplementation. Regular hospital review during treatment did not reveal interstitial lung disease. In January 2022, due to resistance to osimertinib, the combination therapy of osimertinib 80 mg/day and savolitinib 200 mg/12 hours was changed after genetic testing. Afterwards, the patient developed chest tightness and shortness of breath, accompanied by cough and yellow and white sticky sputum. Chest tightness and shortness of breath then progressively worsened, and paroxysmal dyspnea occurred at night. He went to the health center and had poor results after anti-infection diuretic treatment. Chest CT (Fig. 1) in an external hospital 1 day before admission showed that the anterior bronchial occlusion of the upper lobe of the left lung, lung cancer with obstructive pneumonia or atelectasis were considered, and the 2 lungs were extensively interstitial changes, and partial consolidation in both lungs was more obvious in the right lung. After going to the emergency department of our hospital, the blood oxygen saturation was 85%, the CRP was 96mg/L, and the EF value was 46% on cardiac ultrasound, and the physical examination found crackles in both lungs, considering severe pneumonia, respiratory failure, and heart failure. She was admitted to the Intensive Care Unit for further treatment.

**Figure 2.**
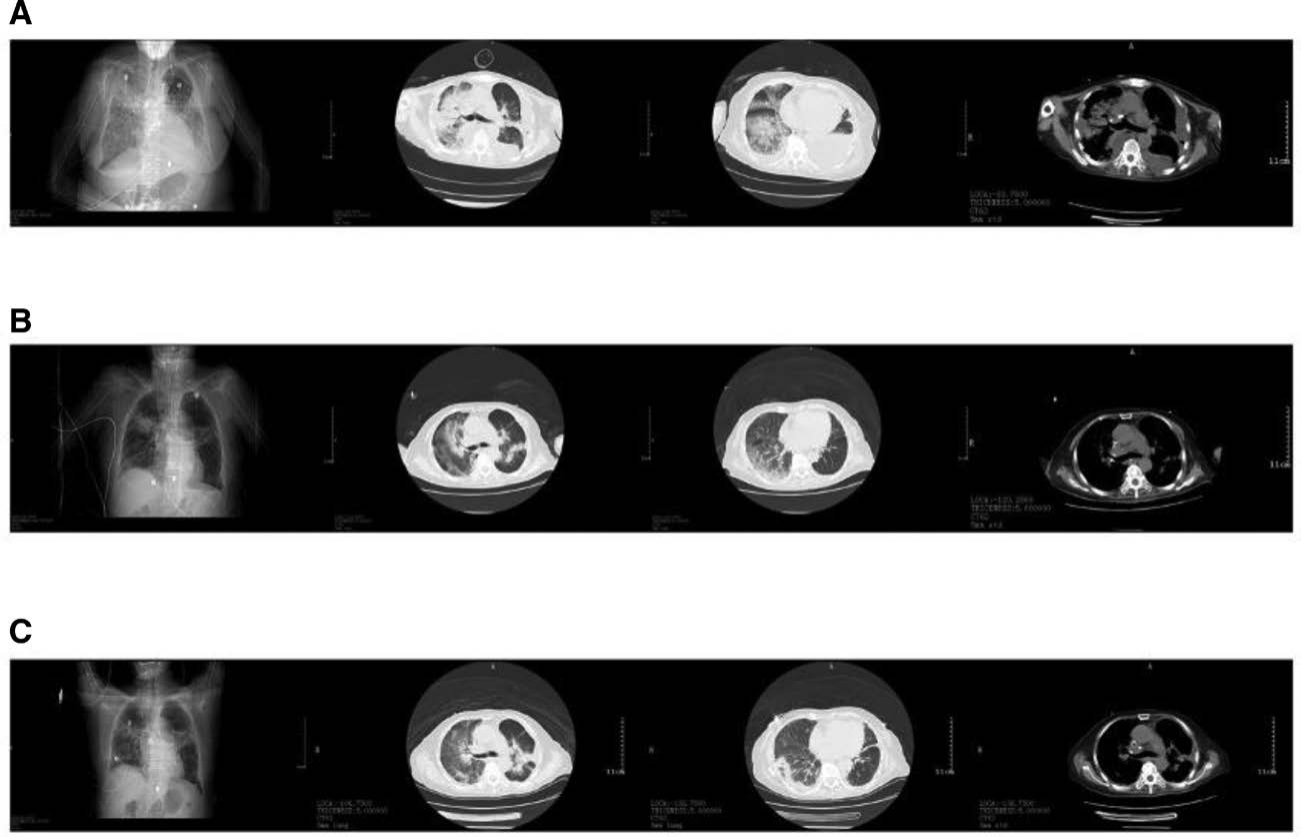
Chest CT during hospitalization (A) Chest CT on the 3rd day after admission (B) Chest CT on the 10th day after admission (C) Chest CT on the 20th day after admission. CT = computed tomography.

After admission, imipenem cilastatin 0.5g/6 hours intravenous infusion was given to anti-infection, and symptomatic treatment such as inhibition of gastric acid, phlegm, reduction of inflammatory reaction, relaxation of bronchi, nutrition of myocardium, etc was given. After admission, the respiratory virus test and the new coronavirus nucleic acid were negative, the CRP was 110mg/L, the N-terminal pro-brain natriuretic peptide (NT-proBNP) was 4490.91pg/mL. Then methylprednisolone 40mg and furosemide 20mg was temporarily given. On the third day after admission, the patient felt chest tightness and shortness of breath aggravated, and the blood gas analysis (high-flow oxygen therapy, oxygen concentration 60%, oxygen flow rate 50L/min) showed that the partial pressure of oxygen was 34mmHg, the partial pressure of carbon dioxide was 36mmHg, and the oxygen saturation was 70.6%. Chest CT (Fig. 2A) showed the progression of 2 pneumonia and increased pleural effusion on both sides, and the left chest closed drainage was performed with B-ultrasound positioning. At the same time, intensive diuresis and cardiotonic therapy are given. Consultation with experts from other hospitals considered the possibility of severe pneumonia, type I respiratory failure, and interstitial pneumonia caused by molecular targeted drugs. After the consultation, all antitumor drugs were discontinued, digoxin was added, methylprednisolone 40mg/12 hours were used, and levofloxacin and caspofungin were added to the anti-infection treatment plan. Repeated blood gas analysis (high-flow oxygen therapy, oxygen concentration 60%, oxygen flow rate 50L/min) showed that the partial pressure of oxygen was 37mmHg, and the bedside ultrasound suggested that the EF value was between 30~35%, and then Milinone was given to enhance myocardial contractility. On the 6th day after admission, the patient was emotional, irritable and gibberish, obvious chest tightness and shortness of breath, heart rate to about 150 beats per minute, and blood oxygen saturation was about 78% lower. Physical examination reveals tachypnea, wheezing and crackles in both lungs. Arterial blood gas analysis (high-flow oxygen therapy, oxygen concentration 60%, oxygen flow 50L/min) showed that the partial pressure of carbon dioxide was 41mmHg, the partial pressure of oxygen was 37mmHg, the lactic acid was 3.5mmol/L, and the NT-proBNP was 8663.11pg/mL. This is followed by endotracheal intubation, mechanical ventilation, diuresis, and norepinephrine to raise blood pressure. Complete bedside bronchoscopy showed that the trachea, carina, upper right, middle lobe segment and upper left bronchial congestion and swelling, the right side was obvious, the left lung tongue segment and lower lobe were mildly engorged, and white sticky sputum was attached to the trachea and each bronchi, which was suctioned. The right lower lobe segment is hyperemic and swollen, and the sputum is thick and difficult to aspirate, after which alveolar lavage is performed and sent for examination. Sputum smears of bacteria, fungi, and Mycobacterium tuberculosis were negative, and alveolar lavage fluid cultures were negative. Subsequent review of chest CT (Fig. 2B) showed that a small amount of pneumothorax on both sides of the new lesion, lung compression of 5-10%, infection of both lungs, pleural effusion was absorbed earlier. Then tracheal intubation and the ventilator were removed.

**Figure 3.**
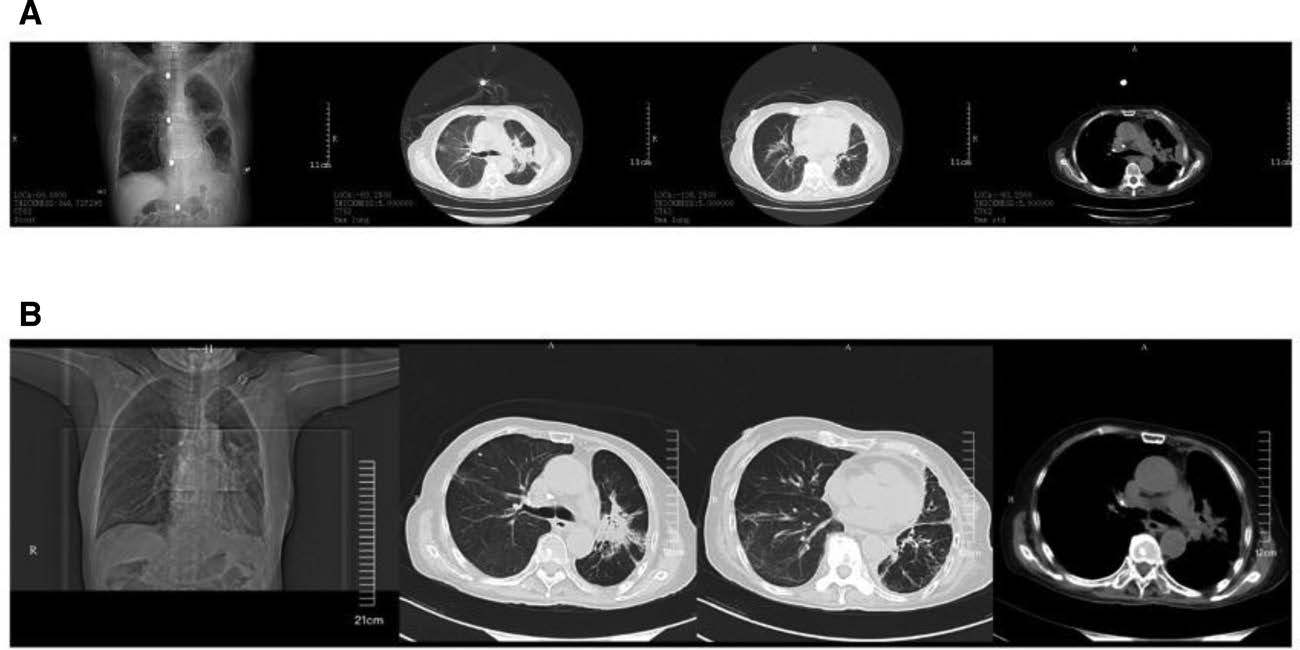
Follow-up chest CT after discharge (A) Chest CT was reviewed in March 2022 (B) Chest CT was reviewed in July 2022. CT = computed tomography.

**Figure 4.**
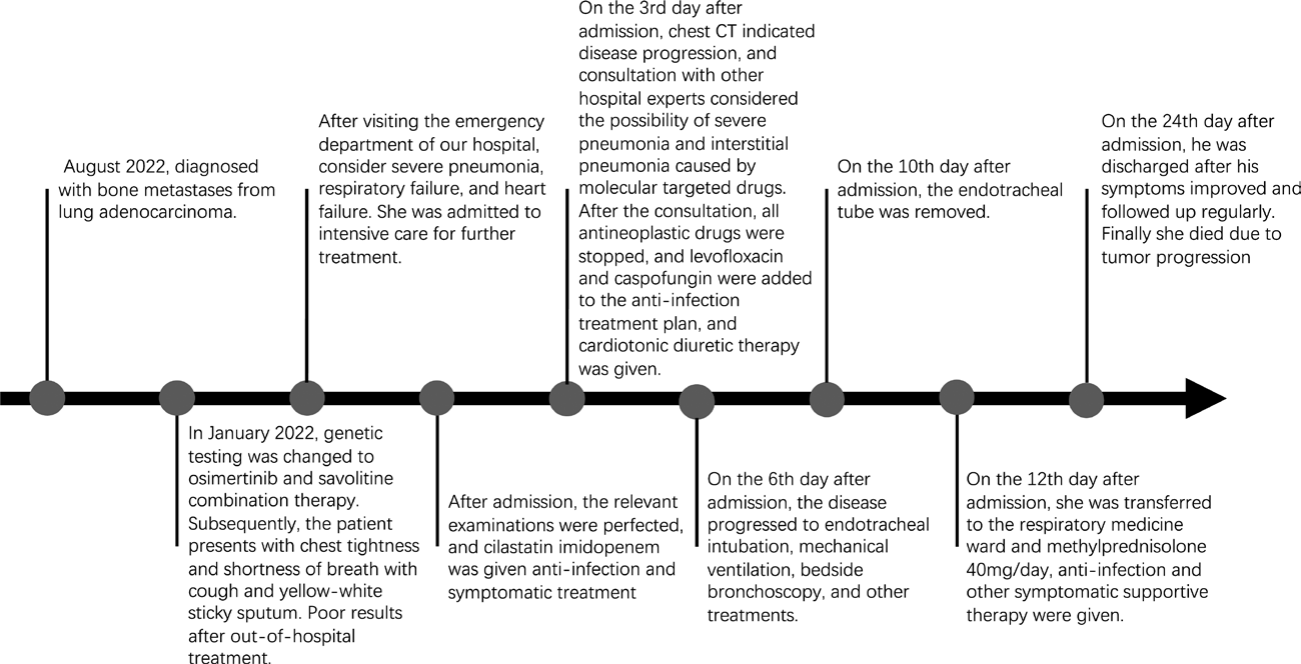
The timeline of the case.

Later, due to the patient poor willingness to treat, he requested further treatment in the respiratory medicine department. After transferring, imipenem-cilastatin was continued to be given anti-infection, methylprednisolone 40mg/day, and other symptomatic supportive therapy were given. After treatment, the patient chest tightness, shortness of breath and cough and sputum were relieved compared with before. The reexamination CRP was 28.28mg/L, NT-proBNP was 1803.68pg/ml, and chest CT (Fig. 2C) was better than before. Afterwards, it was adjusted to cefoperazone-sulbactam for anti-infection, prednisone 20mg/day, diuretic and potassium supplementation. The patient was discharged from the hospital after her symptoms were relieved and her condition was stable. After discharge, she continued to receive oral prednisone 10 mg/day, cardiotonic diuretic therapy and outpatient follow-up. Repeat chest CT in March and July 2022 (Fig. 3) showed that interstitial pneumonia disease (ILD) was better than before. She passed away due to tumor progression. The important timeline of this case is shown in Figure 4

## 3. Discussion

### 3.1. Etiology and mechanism of drug-induced interstitial lung disease

Interstitial lung disease is a group of heterogeneous diseases with diverse etiologies, which can be roughly divided into idiopathic, autoimmune-related, exposure-related (including iatrogenic), interstitial lung disease with cysts or air cavity filling, and sarcoidosis. Chemotherapy, immune checkpoint inhibitors, biologics, etc are common causes of drug-induced interstitial pneumonia disease (DI-ILD).^[9]^ DI-ILD involves 2 mechanisms that may be interdependent: one is direct dose-dependent toxicity and the other is immune-mediated. Cytotoxic lung injury may result from direct damage to lung cells or alveolar capillary endothelium, and most responses in immune-mediated DI-ILD may be T cell-mediated.^[10]^

### 3.2. Clinical application of savolitinib in lung cancer

Savolitinib is a MET-TKI approved in China for the treatment of metastatic NSCLC with MET exon 14 skipping changes that progress after platinum-based chemotherapy or cannot tolerate platinum-based chemotherapy, based on the results of a pivotal phase II trial in NSCLC/lung sarcoma-like carcinoma.^[11]^

Currently, TATTON studies have shown the feasibility of combining osimertinib 80 mg with savolitinib at identified tolerable,^[12]^ and on-treatment ctDNA dynamics can predict clinical outcomes and may provide an opportunity to inform earlier decision-making.^[13]^ Meanwhile, the FLOWERS study also showed better perspectives on the efficacy and safety of EGFR-TKI plus MET-TKI combination therapy in patients with de novo MET-amplified/over-expressed, EGFR-mutant positive, treatment naïve, advanced NSCLC and offer a meaningful guidance in clinical practice.^[14]^

### 3.3. Case reports of interstitial pneumonia disease Induced by EGFR-TKI or MET-TKI

The overall incidence of EGFR-TKIs-related lethal toxic reactions is 1.33%,^[15]^ from the current post-marketing safety report of EGFR-TKI, afatinib has a higher proportion of adverse reactions in causing skin and gastrointestinal reactions, gefitinib seems to be prone to ILD and liver damage, and QT interval prolongation seems to be more frequent in patients treated with osimertinib. In clinical trials, the incidence of ILD in the osimertinib group was 3.9%.^[16]^

There have been no clear reports of MET-TKI causing interstitial lung disease. Ground-glass opacity of the lungs during treatment with MET-TKI (tepotinib) has been reported, which resolves spontaneously with drug discontinuation.^[17]^ Savolitinib is a MET-TKI launched in 2021, and common adverse reactions are peripheral edema, nausea, elevated alanine transaminase, elevated aspartate transaminase, vomiting, hypoalbuminemia, and decreased appetite. Although interstitial lung disease is rare, interstitial lung disease has been reported to occur after the use of TKI for lung cancer, and the onset is often rapid; Interstitial lung disease occurred in 2.2% to 4.5% of METex14-jumping NSCLC patients treated with capmatinib, tepotinib, or crizotinib, and 0.3% to 0.5% died.^[8]^ Whether EGFR-TKI combined with MET-TKI increases the incidence of interstitial lung disease needs more further study.

### 3.4. Diagnosis and treatment of drug-induced interstitial lung disease

The diagnosis of DI-ILD usually depends on a clear temporal association between exposure to the causative agent and the onset of respiratory signs and symptoms.^[10]^ Important components of the diagnostic process include physical examination and careful patient history, vital signs measurements (especially respiratory rate and arterial oxygen saturation), relevant laboratory tests, respiratory function tests using spirometry, and lung carbon monoxide diffusion capacity and computational tomography/imaging. Because the clinical and radiological signs of DI-ILD often mimic pneumonia or interstitial lung disease, differential diagnosis is important, including microbiological and serological testing to exclude or confirm the infection.^[18]^

For drug-induced interstitial lung disease, discontinuation of the suspect drug is key to treatment. Glucocorticoids are generally used in patients with disability or disease progression and may be effective in critically ill patients, but high-quality clinical studies are lacking. For antineoplastic drugs, discontinuation requires careful consideration of the risks and benefits and availability of alternative therapies. Whether to reuse the same drug after pulmonary toxicity caused by a previous drug must be a comprehensive decision based on the specific situation, severity of response, and availability of alternative therapies, with relapse DI-ILD occurring in approximately one-third of retreated cases.^[19]^

## 4. Summary

Savolitinib is a MET-TKI marketed in 2021, and pulmonary toxicity can be observed in animal experiments, but it is unclear whether ILD is its adverse reaction. From this case, we can know that osimertinib combined with savolitinib may cause interstitial pneumonia. However, since this study is only a case report, whether ILD in this case is caused by savolitinib itself or the combination of 2 drugs increases the risk of ILD caused by EGFR-TKI is unclear, and further confirmation is needed in high-quality clinical studies. DI-ILD has a high mortality rate, so early recognition and intervention are particularly important. This case suggests that corticosteroids may play an important role in treatment.

## Author contributions

**Investigation:** Songgao Lou.

**Writing – original draft:** Yikuan Shen.

**Writing – review & editing:** Jiansong Zhang.
